# Optimization of Wavy-Channel Micromixer Geometry Using Taguchi Method †

**DOI:** 10.3390/mi9020070

**Published:** 2018-02-06

**Authors:** Nita Solehati, Joonsoo Bae, Agus P. Sasmito

**Affiliations:** 1 Department of Industrial and Information Systems Engineering, Chonbuk National University, 567 Baekje-daero, Deockjin-gu, Jeonju 54896, Korea; nita.solehati@gmail.com; 2 Department of Mining and Materials Engineering, McGill University, Adams Building #115, 3450 University Street, Montreal, QC H3A 2A7, Canada; ap.sasmito@gmail.com or agus.sasmito@mcgill.ca

**Keywords:** geometrical design, micromixer, optimum, Taguchi, wavy-channel

## Abstract

The micro-mixer has been widely used in mixing processes for chemical and pharmaceutical industries. We introduced an improved and easy to manufacture micro-mixer design utilizing the wavy structure micro-channel T-junction which can be easily manufactured using a simple stamping method. Here, we aim to optimize the geometrical parameters, i.e., wavy frequency, wavy amplitude, and width and height of the micro channel by utilizing the robust Taguchi statistical method with regards to the mixing performance (mixing index), pumping power and figure of merit (FoM). The interaction of each design parameter is evaluated. The results indicate that high mixing performance is not always associated with high FoM due to higher pumping power. Higher wavy frequency and amplitude is required for good mixing performance; however, this is not the case for pumping power due to an increase in Darcy friction loss. Finally, the advantages and limitations of the designs and objective functions are discussed in the light of present numerical results.

## 1. Introduction

Recent advances in micro-reactor technologies have enabled chemical processes and pharmaceutical industries to produce high quality products due to their ability to control the extreme/unusual reaction environments, such as highly exothermic or explosive chemical reaction, highly viscous fluids which are difficult to mix in larger scale mixing equipment, etc. There are many other advantages of micro-reactor technology, such as higher transport rate, safer environment, compact design and simpler process control. Despite its advantages, micro-reactor also has limitations, especially when large throughput product in industrial scale is desired, whilst a small size micro-reactor can only produce a small amount of yield, and enlarging the micro-reactor size (scaling-up) decreases the product quality. One way to increase the product output is by numbering-up the micro-reactors into several modulars; the modular comprises several mixing zones (mixers) and reaction zones (reactors). However, one of the major drawbacks of this design is the flow uniformity, for which the reactant flow may not be uniformly distributed throughout each micro-channel, which causes non-uniform product quality. Liu et al. [1] proposed the structural bifurcation of the flow channel to improve flow uniformity throughout the micro-reactor/micro-mixer modular. 

The conventional micro-mixer design typically uses a T-junction with straight micro-channel: the T-junction consists of at least two inlets for the reactant to enter and further mix in the straight micro-channel. This design is widely used in chemical and pharmaceutical industries due to its ease of manufacture. However, this design has poor mixing quality, as small-scale mixing depends mainly on the molecular diffusion. Thus, a relatively long channel and higher pumping power is required to achieve desired mixing, which can be impractical. Several designs have been proposed by many researchers (see [2,3,4,5,6] for reviews of these) to improve the mixing quality. However, most of the proposed passive mixer designs require complex geometrical structures which are difficult/expensive to manufacture. Active mixers, on the other hand, have been proven to significantly enhance mixing and reaction rate; for example, the use of acoustic waves in sidewall sharp-edges [7,8], acoustic waves on Y-cut 128° LiNbO_3_ [9] and the use of lateral acoustic transducer in micromixer [10]. Despite its advantages, the active mixer requires additional equipment, such as a wave generator, external piezoelectric buzzer and so forth, which adds capital and operating costs as well as complexity. For the industrial scale of cheap and mass-produced chemical product, active mixers increase the total production cost and may not be preferred. Hence, a simple, cheap, reliable and high performance passive micromixer is desired by such industry.

Currently, there are many available micromachining methodologies for rapid microchannel manufacturing; these include the casting of laser ablation, hot embossing, polydimethylsiloxane (PDMS), micropowder blasting, stereolithography, micromilling, and lamination. Lamination of polymeric films is another fast prototyping process for thermoplastic-based microfluidic devices. However, as it is mostly performed by laser cutting and lamination of thin layers of plastic, it requires expensive equipment and has a high maintenance cost. While the most advanced micomanufacturing technologies enable the creation of complicated three-dimensional microchannel structures, they are, however, only feasible to be implemented for high value product in their current stage due to their complication processes and expensive cost. Tonkovich et al. [11] showed that the most economical method for mass manufacturing of microchannel reactors is the stamping method; however, this method is unable to create complex geometry in three-dimensional shapes as proposed by many researchers to enhance mixing performance. Recently, the stamping method has been successfully used to fabricate cooling channels [12], microfluidic on sheath flow [13], and gas channels in proton exchange membrane fuel cells [14]. Choi et al. [15] showed that PDMS stamp can be used to fabricate micro- and nanopatterns. Thus far, the stamping method has been proven to be very economical for this purpose. The only foreseen possible drawback is the precise control of the stamping force that might not be uniform throughout the channel, and thus may create a slight error in providing the desired uniform channel height.

Numerous experimental and numerical studies to evaluate mixing enhancement have been performed. Hossain et al. [16] proposed two-layer serpentine crossing channels for mixing enhancement at low Reynolds numbers. In another study, Hossain and Kim [17] introduced the concept of the three-dimensional serpentine split-and-recombine (SAR) microchannel using a series of “OH”-shaped segments which showed a mixing index of 0.884 at Re = 30. Ahmed and Kim [18] evaluated the effect of geometrical parameters on an electro-osmotic micromixer with heterogeneous charged surface patches. Xie and Xu [19] simulated an oscillating feedback micromixer comprising an inlet channel, two Coanda steps, a divergent chamber, a splitter, two feedback channels, and an outlet channel was designed considering the Coanda effect. The results indicated that the mixing efficiency increased with the increase of Reynolds number, and a mixing efficiency of 75.3% could be achieved at Re = 100. Despite the wide range of studies performed to come up with the most efficient micromixer geometry, most of the proposed geometries have a difficult shape to be mass-manufactured.

In previous work [20], we introduced a micro-mixer with wavy structure for improved mixing performance but which was easy to manufacture by using the stamping method, similar to conventional micro-mixer design. We also investigated the details of the flow and mixing behaviour in this type of new micro-mixer design [21]; it was found that our new design of micro-mixer with wavy structure has superior performance as compared to conventional design and has comparable performance with micro-mixers with complex geometrical structures which are difficult and/or expensive to manufacture. However, for the best and better performance of our micro-mixer design with wavy structure, optimization of geometrical design is required, which is the theme of this work.

In previous work, we had successfully utilized the computational fluid dynamics (CFD) approach together with the Taguchi statistical method to optimize the performance of liquid-cooled fuel cells [22] and open-cathode fuel cells [23]. To continue our work in the area of numerical micro-mixer design and optimization using Taguchi statistical method, the aim of the study presented is twofold: (i) to optimize geometrical design—wavy frequency, wavy amplitude, micro-channel width and height; (ii) to evaluate the objective function of optimization with regards to the mixing performance (mixing index), parasitic load (pumping power) and figure of merit for different industrial application.

The layout of the paper is as follows. First, the model development using computational fluid dynamics (CFD) is introduced; it comprises conservation equations of mass, momentum and species for mixing. The mathematical model is then solved numerically utilizing finite-volume-based CFD software ANSYS Fluent 16 (Ansys, Inc., Canonsburg, PA, USA). The mixing performance of the conventional T-junction design is compared with wavy channel and complicated channel design along with parametric study on geometrical parameters. The Taguchi statistical method is then employed to study the sensitivity of each design parameter. Optimum parameters are then calculated based on the mixing performance, pumping power and figure of merit defined later. Finally, advantages and limitations of the design are highlighted, and conclusions are drawn based on the results presented.

## 2. Model Development

**Figure 1:**
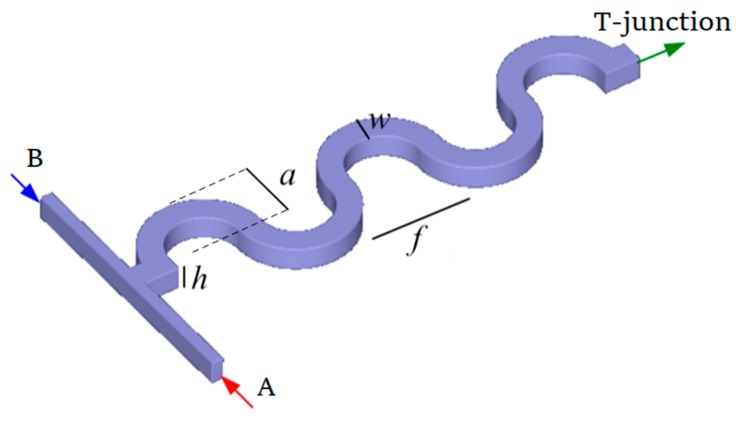
Schematic of micromixer T-junction with wavy structure.

The physical model (see Figure 1) comprises a micro-wavy-channel design for which liquid A enters the channel from the right inlet (red arrow in Figure 1), while liquid B flows from the left inlet (blue arrow in Figure 1). Liquids A and B mix in the opposing streams in a T-junction. The channel height (*h*), width (*w*), wavy frequency (*f*) and wavy amplitude (*a*) are varied according to the Taguchi array. For comparison purposes, we keep the length the same for all cases.

### 2.1. Governing Equations

The conservation equations of mass, momentum and miscible species are given by

(1) ∇·ρu=0

(2) $$\nabla \cdot \rho u\;\times \;u=-\nabla p+\nabla \cdot \left[\left\{\mu \left(\nabla u+{\left(\nabla u\right)}^{T}\right)\right\}\right]$$

(3) $$\nabla \cdot \left(\rho u\omega_{i}\right)=-\nabla \cdot \left(\rho D_{i}\nabla \omega_{i}\right)$$

In the above equations, *ρ* is the fluid density, ***u*** is the fluid velocity, *p* is the pressure, *μ* is the dynamic viscosity, *ω_i_* is the mass fraction of species *i*, *D_i_* is the diffusion coefficient of species *i*.

The mixing performance is evaluated using mixing index, defined as

(4) $$\tau^{2}=\frac{1}{n}\sum_{i=1}^{n}{\left(\omega_{i}-\omega_{\infty }\right)}^{2}$$

(5) $$M_{i}=1-\sqrt{\frac{\tau^{2}}{\tau_{max}}}$$

where *τ* indicates the variation of concentration for each cross section, *τ_max_* is the maximum variance over the range of data, *n* is the number of sampling points inside the cross-section, *ω_i_* is the mass fraction at sampling point *i*, *ω*_0_ is the initial concentration, *ω_∞_* is the concentration at infinity, and *M_i_* is the mixing index. The mixing index is unity for complete mixing, and zero for no mixing. The values at the sampling points were obtained by interpolation with the values from adjacent computational cells.

To ensure the fidelity of comparison for both micromixer designs, the concept for figure of merit (FoM) is introduced to evaluate the effect of Reynolds number and the effect of geometry on the pressure drop and mixing performance. FoM is defined as the ratio of the mixing index per unit pressure drop required, given by:

(6) $$\mathrm{FoM}=\frac{M_{i}}{∆p}$$

### 2.2. Boundary Conditions

The boundary conditions for the flow inside the micro-channel T-junction are as follows:

Right inlet: liquid A is introduced to the channel; we prescribe inlet velocity and species mass fraction.

(7) $$u=U_{A},\;\omega_{A}=1,\omega_{B}=0$$

Left inlet: liquid B enters the channel; constant inlet velocity and species mass fraction are prescribed.

(8) $$u=U_{B},\;\omega_{A}=0,\omega_{B}=1$$

Outlet: we specify the pressure and stream-wise gradient of the temperature, and species mass fraction is set to zero. The velocity is not known a priori but needs to be iterated from the neighboring computational cells.

(9) $$p=p_{out},\;n\cdot \nabla \omega_{i}=0$$

At the walls: we specify no slip condition and no species flux at the channel wall.

(10) $$u=0,\;\nabla \omega_{i}=0$$

### 2.3. Taguchi Statistical Method

The Taguchi method is a well-known statistical method developed by Genichi Taguchi. It is a powerful engineering tool for experimental optimization and one of the most well-known robust design methods. Generally, it is used to find the sensitivity of each parameter and determine the optimum combination of the design factors [24,25]. Here, we have four key parameters, e.g., wavy frequency, wavy amplitude, channel width and channel height, with three level values for each parameter. An L_9_ orthogonal array (OA) was employed in the experiment matrix, as shown in Table 1. It is worth mentioning that if one would like to investigate the effect of combination of parameters and optimize the design without the Taguchi statistical method, the total number of simulations would have been prohibitive; for example, as we have four parameters and three levels, the total number of simulation is 4^3^ = 64 simulations. In a wavy micromixer, the mesh size needs to be very fine and computationally expensive. The statistical method, on the other hand, can be used to effectively reduce the number of simulations and computational cost, evaluate the interaction of parameters and optimize the design.

In this paper, we evaluate the objective function of the optimum parameters based on the mixing index, Equation (5), pumping power (pressure drop) and figure of merit, Equation (6); therefore, we evaluate the signal-to-noise (S/N) ratio based on the-larger-the-better for mixing index and FoM:

(11) $$S/N=-10log_{10}\left(\frac{1}{n_{r}}\sum_{i=1}^{n_{r}}\frac{1}{Y_{i}{}^{2}}\right)$$

Whereas, for pumping power, we calculate the signal-to-noise (S/N) ratio based on the-smaller-the-better:

(12) $$S/N=-10log_{10}\left(\frac{1}{n_{r}}\sum_{i=1}^{n_{r}}Y_{i}{}^{2}\right)$$

Once the optimum combination of each parameter has been determined, we verify the predicted results from Taguchi method with CFD results. The confidence interval (CI) of the estimated value is calculated by:

(11) $$\mathrm{CI}=\sqrt{F_{\alpha ,v_{1},v_{2}}V_{\mathrm{ep}}\left(\frac{1}{n_{\mathrm{eff}}}+\frac{1}{r}\right)}$$

where $F_{\alpha ,v_{1},v_{2}}$ is the F-ratio required, *v*_1_ is the number of degree of freedom of the mean, *v*_2_ is the number of degree freedom of the error, *V*_ep_ is the error of variance, *r* is the sample size in the confirmation test, and *n*_eff_ is the effective sample size, defined as

(12) $$n_{\mathrm{eff}}=\frac{N}{1+{\mathrm{DOF}}_{\mathrm{opt}}}$$

where *N* is total number of trials and DOF_opt_ is the total degree of freedom that are associated with items used to estimate $\eta_{\mathrm{opt}}$.

## 3. Numerics

The computational domains (see Figure 1) were created in AutoCAD 2010 (Autodesk, Inc., San Rafael, CA, USA); the commercial pre-processor software Gambit 2.3.16 (Ansys, Inc., Canonsburg, PA, USA) was used for meshing, labeling boundary conditions and to determine the computational domain. Three different mesh designs—1 × 10^7^, 2 × 10^7^ and 4 × 10^7^—were implemented and compared in terms of the local pressure, velocities, species mass fractions and temperatures to ensure a mesh independent solution. We found that the mesh numbers around 2 × 10^7^ give about 1% deviation compared to a much finer mesh size of 4 × 10^7^; whereas the results from the mesh size of 1 × 10^7^ deviate up to 10% as compared to those from the finest mesh design. Therefore, a mesh consisting of around 2 × 10^7^ elements was found to be sufficient for the numerical experiments: a fine structured mesh was used near the wall to resolve the boundary layer and an increasingly coarser mesh in the middle of the channel in order to reduce the computational cost. The aspect ratio of the boundary layer mesh is 2, with a thickness of about 10 to 20% of the total height/width. The detail validation of the mesh independence test was published in the earlier paper [20]; for the sake of brevity, we do not repeat in this paper.

The equations were solved with the well-known semi-implicit pressure-linked equation (SIMPLE) algorithm, first-order upwind discretization and algebraic multi-grid (AMG) method. As an indication of the computational cost, it is noted that, on average, around 5000–10,000 iterations are needed for convergence criteria for all relative residuals of 10^−9^; this takes around two days on a computer cluster with 16 processors and 20 GB of random access memory (RAM).

The key operating parameters are then analyzed using the Taguchi statistical method in Minitab 14 software. A variance analysis (ANOVA) was performed in order to see the sensitivity of each parameter, to determine the optimum combination of operating parameters and to evaluate the confidence levels between the Taguchi prediction and CFD results.

## 4. Results and Discussion

The numerical simulations were carried out for typical conditions found in micro-channel T-junctions; the base-case conditions together with the physical parameters and geometric parameters are listed in Table 2 which were chosen based on the typical microchannel geometries. Model verification was carried out in earlier publication [20] and for the sake of brevity is not repeated here. In the following, sensitivity analysis of each design parameter is investigated from the response of signal-to-noise ratio of OA. Optimum design parameters are then examined based on the mixing index, pressure drop (pumping power) and figure of merit (FoM).

### 4.1. Effect of Channel Geometries

**Figure 2:**
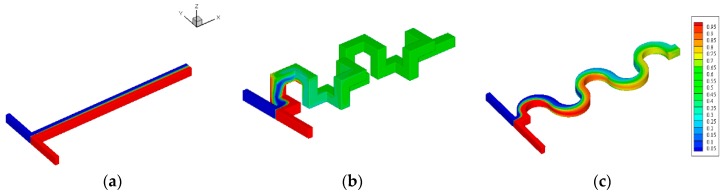
Mixing performance of micromixer T-junction designs. (**a**) Conventional straight; (**b**) Complex 3D serpentine; (**c**) Wavy microchannel.

One of the key factors that determine the mixing and reaction performance is the geometric design of the channel. This study examines three different micro-channel T-junction geometries: straight, complex 3D serpentine channel and wavy channel with same total length and fluid velocity. Since the mixing is directly linked to the flow behavior and total mixing time, it is of interest to investigate the flow patterns inside the channel. Previous work on wavy microchannels [20] showed that the presence of centrifugal force due to curvature leads to significant radial pressure gradients in the flow core region. In the proximity of the inner and outer walls of the coils, however, the axial velocity and the centrifugal force approach to zero. Hence, to balance the momentum transport, secondary flow should develop along the outer wall. This is indeed the case, as can be seen in Figure 2, where the secondary flows present in the complex 3D serpentine channel and wavy channel (Figure 2b,c). This, however, is not the case for the straight T-junction, as a fully developed flow exists inside the channel. It is noted that, at this particular inlet Reynolds number (~10, Re = *ρUDh/μ*), the secondary flows appear as two pairs in wavy channel as the wavy structure inverted the secondary flow direction.

The presence of secondary flow with higher velocities toward the outer wall of the complex 3D serpentine and wavy channels is expected to have direct impact on mixing characteristics. This can be inferred from Figure 2, which presents local mass fraction distribution of liquid mixing over the cross sections of various channel designs. Here, several features are apparent; foremost is that, for the same total mixing time, the complex 3D serpentine channel yields the best mixing performance with mixing index of nearly perfect 1 (0.999). The conventional straight channel T-junction, on the other hand, performs the worst among the others, with a mixing index of 0.071. On closer inspection, we note that the mixing is very poor for the straight channel T-junction as the liquid is hardly mixing throughout the channel outlet, as can be seen in Figure 2a, while the mixing performance of the wavy microchannel lies between the conventional straight T-junction and complex 3D serpentine channel with mixing index of 0.476. This indicates that the wavy microchannel has the potential to be used as a passive mixer, as the manufacturing cost and processes are much simpler than that of the complex 3D serpentine design; of course, further geometry optimization is required for best performance.

### 4.2. Mixing Performance

This study examines the mixing performance based on OA of the Taguchi method, which is tabulated in Table 3 and for which the best mixing is achieved by design number 9 and the worst performance is given by design number 2. The sensitivity of each parameter is then analyzed by employing analysis of variance (ANOVA). Typically, for high quality and expensive chemical and/or pharmaceutical products, product quality—in this regard reflected by mixing quality—is of paramount importance. Thus, the objective function of our optimization is based on the mixing index. The higher the mixing index, the better the mixing quality.

**Figure 3:**
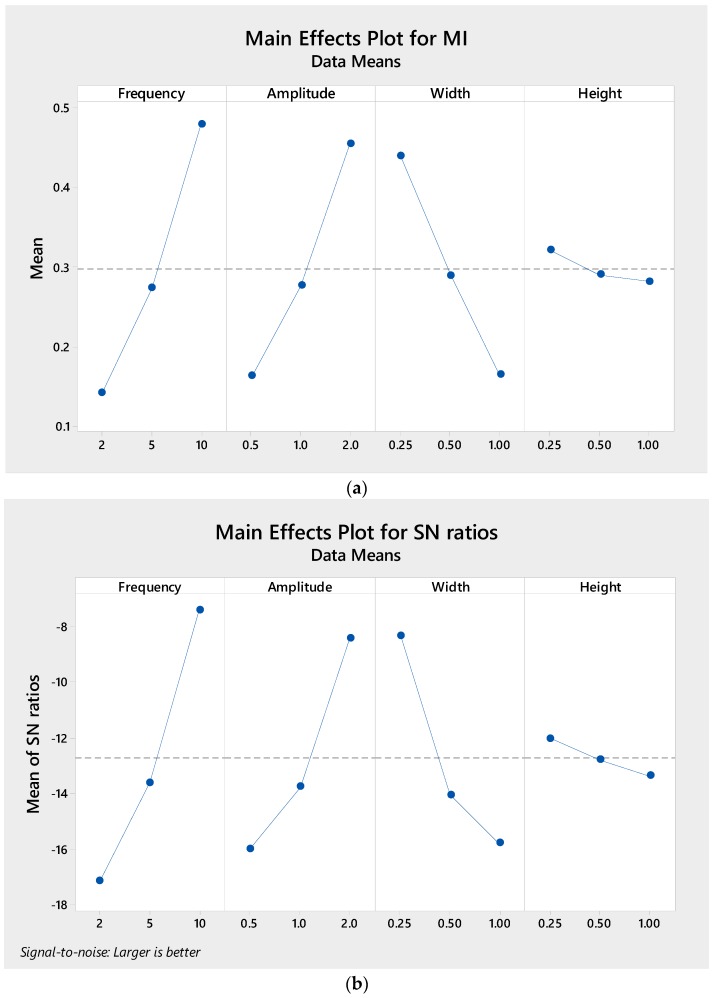
Taguchi results of (**a**) Mean and (**b**) signal-to-noise (S/N) response graph for mixing performance.

Earlier work [12] showed that wavy amplitude and frequency play a significant role in the mixing performance; this is indeed the case, as can be inferred from Figure 3, where wavy frequency results in the most significant parameter influencing the mixing performance, followed by wavy amplitude. Higher wavy frequency and longer wavy amplitude improve mixing performance. This can be adequately explained by the fact that, at higher wavy frequency and longer wavy amplitude, the secondary flow generated by curve geometry is stronger, which enhances mixing. Further, increasing frequency and amplitude increases the total microchannel length, which in turn increases the residence time of the fluid mixing. On the other contrary, the width of the microchannel has a less significant effect, while microchannel height has the least significant effect on the mixing performance. We note that mixing performance improves as the width and height are decreased. This is attributed to the smaller channel dimension which reduces the diffusion path between two fluids to penetrate each other and mix.

**Figure 4:**
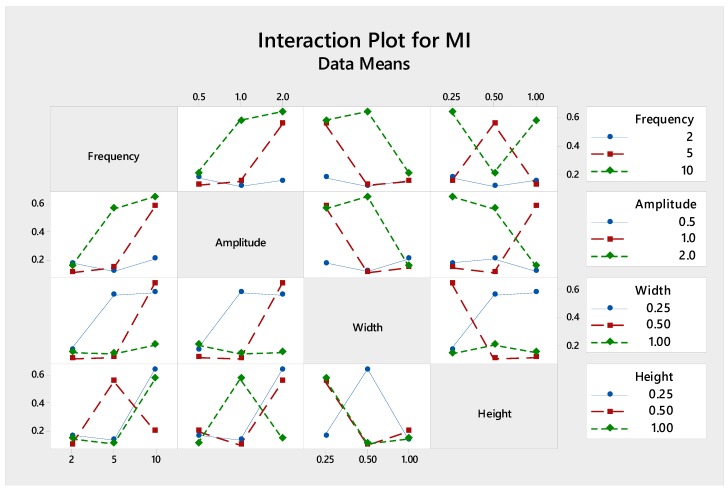
The interactions of various parameters with respect to mixing index.

Figure 4 shows an interaction plot for each factor, for which parallel plot denotes no interaction while crossing indicates significant interaction. Interestingly, although channel height yields the least significant individual effect to the mixing performance, it shows the strongest interaction with other parameters, as shown by the crossing lines in Figure 4. It is worth mentioning that channel height determines the characteristic length of the channel which defines the Reynolds number and, thus, the mixing flow regimes, i.e., segregated, vortex, engulfment and chaotic flow, and mixing performance [12].

Thus far, the sensitivity of each parameter has been examined. Now, the optimum combination of design parameters is determined. We further predict the optimum mixing performance using the Taguchi method and run the confirmatory test from CFD model. The results are depicted in Table 4 for which good agreement between the Taguchi prediction and CFD mode was obtained within the maximum error of less than 6%, which is sufficient for engineering purposes. The optimum mixing index for the optimized design is found to be 0.8.

### 4.3. Pumping Power

Generally, in cheap and mass production of chemical products, production cost becomes the most significant factor. One of the factors that constitute the production cost is the pumping power required to drive the flow mixing. Here, we evaluate the pumping power by looking at the pressure drop required. In essence, to reduce production cost, the pumping power which is represented by pressure drop should be as low as possible. The results for pressure drop required of OA are summarized in Table 3, column 3. It is seen that the highest pressure drop required is in design number 9, which is about three order-of-magnitudes higher than that of design number 3 which yields the lowest pressure drop. It is important to note that design number 9 has higher mixing performance with the expense of a much higher pressure drop. Thus, for cheap and mass production chemical product, this design seems to be not attractive, as they would prefer to implement the design with the lowest pumping power to save power/electricity cost.

**Figure 5:**
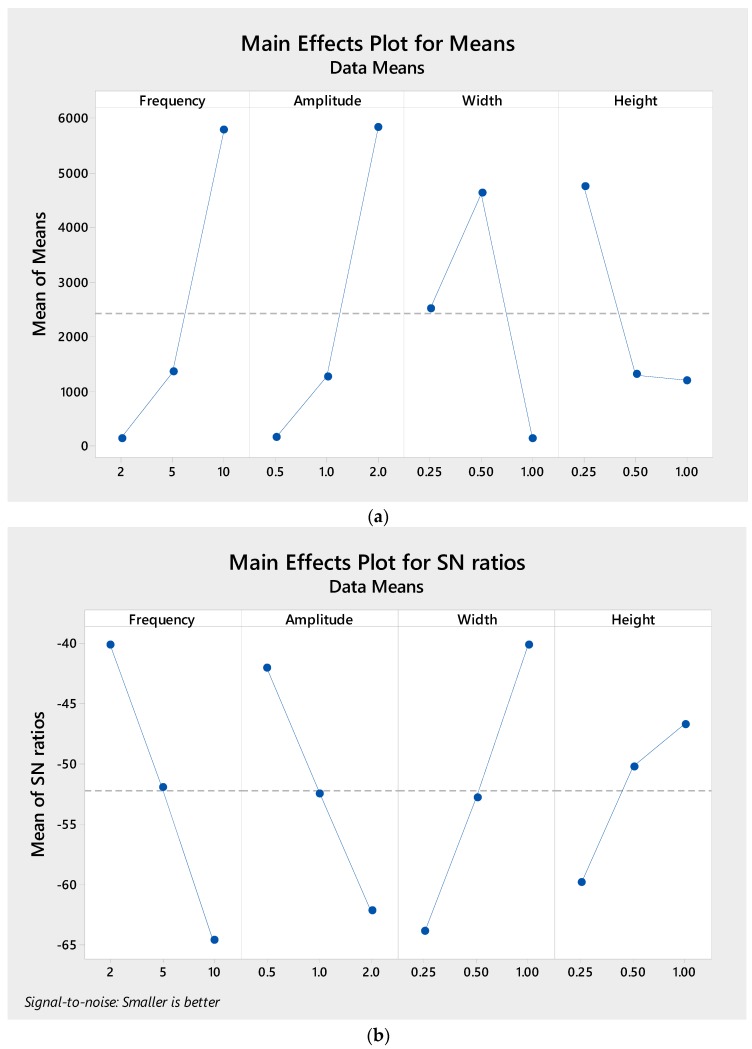
Taguchi results of (**a**) Mean and (**b**) S/N response graph for pumping power.

With regard to the pumping power, the sensitivity of each design parameter is evaluated. Figure 5 shows the behavior of each parameter, which is somewhat different than when it was evaluated in term of mixing performance. Here, several features are apparent; foremost among them is that the low pressure drop can be obtained at low wavy frequency, low wavy amplitude, longer width and longer height, which is opposite to mixing performance. This is due to the fact that reducing frequency and amplitude reduces flow resistance throughout the microchannel as the tube length has a proportional relation with pressure drop due to the increase in Darcy friction loss (Δ*p = f*_D_ *× L/D × ρU*^2^*/*2).

**Figure 6:**
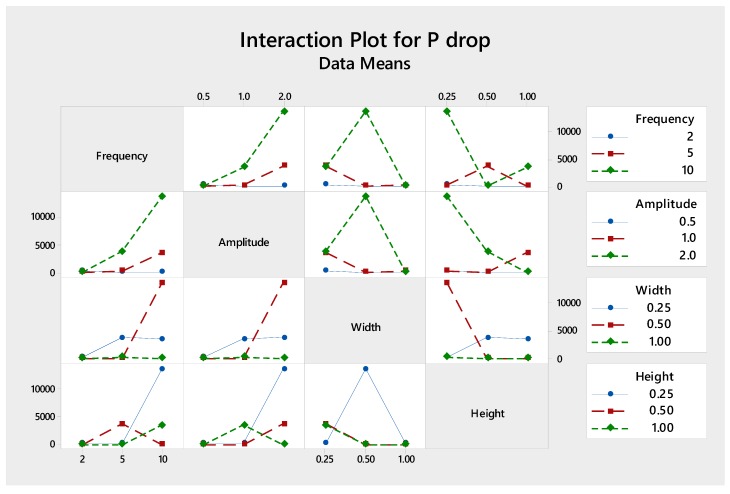
The interactions of various parameters with respect to pumping power.

Looking at the interaction of each factor in Figure 6, it is found that significant interaction is obtained between channel height and frequency, channel width and amplitude, and channel height and width, which is reflected by interaction of *L* and *D* in Darcy friction loss equation.

Now, the combination of optimum factors is evaluated to get the design with the lowest possible pumping power. Table 4 depicts the optimum (minimum) pumping power required. It is noted that, at optimum condition, the pumping power required is 21.75 Pa which is about half than that of design number 3. The level of confidence from Taguchi prediction is observed to be 91.8% which is good enough for engineering design.

### 4.4. Figure of Merit

So far, we have evaluated the micro-mixer based on mixing performance and pumping power separately. To balance and take into account both effects, we consider the figure of merit, which basically is defined as mixing performance per unit pumping power. Table 3 shows the FoM of OA condition. We note that the highest FoM is achieved by design number 3 due to reasonable mixing performance with the lowest pressure drop requirement; whereas the lowest FoM is seen in design number 9, since the pumping power is very high (about three orders-of-magnitude) compared to design number 3.

**Figure 7:**
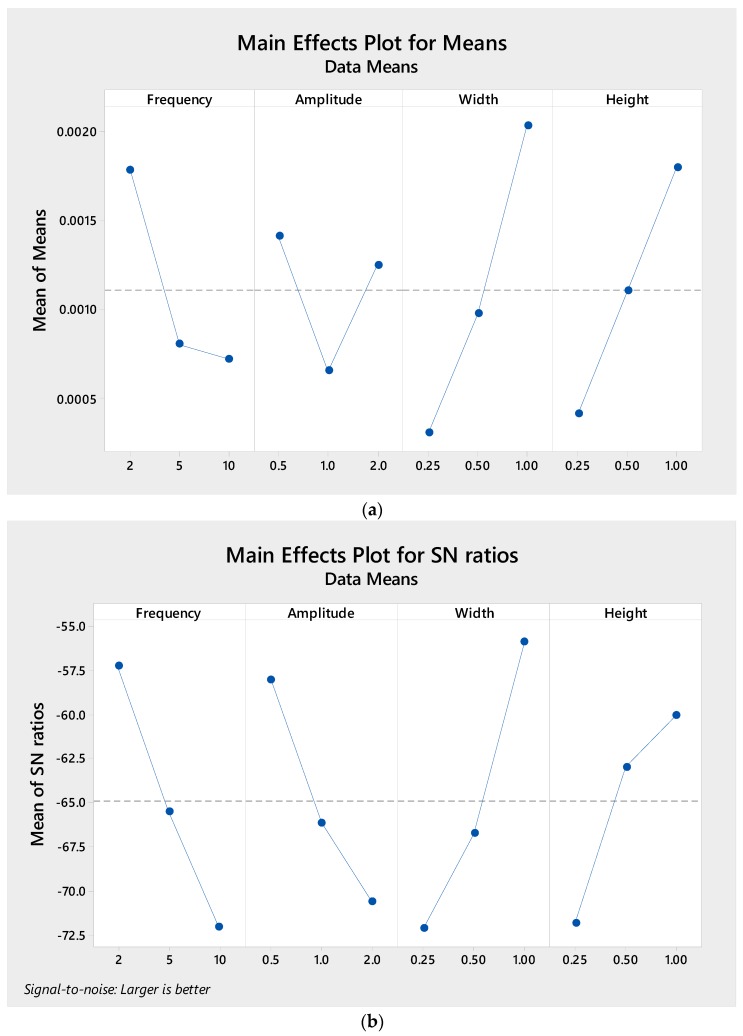
Taguchi results of (**a**) Mean and (**b**) S/N response graph for figure of merit (FoM).

**Figure 8:**
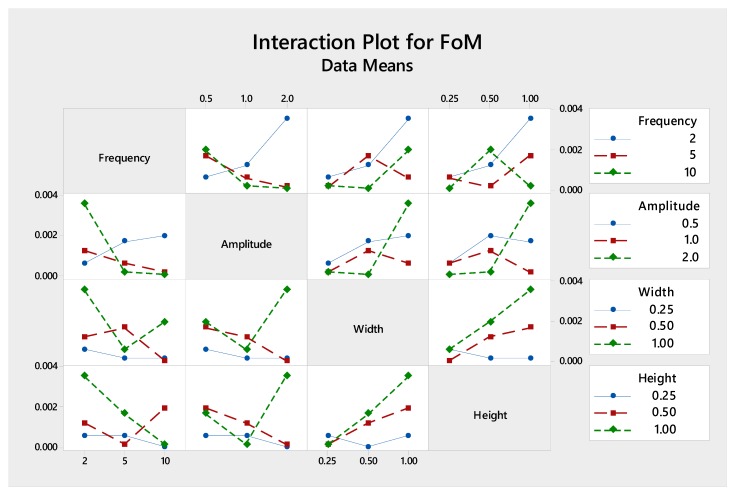
The interactions of various parameters with respect to figure of merit (FoM).

Looking further to the sensitivity response of *S*/*N* ratio for each parameter in Figure 7, it reveals that the trend is similar to that in pumping power: lower wavy frequency, shorter wavy amplitude, longer microchannel width and longer microchannel height. Turning our attention to the interaction of each factor, Figure 8 depicts a significant interaction between frequency and amplitude with all parameters at different levels, while the interaction between channel width and height is marginal.

Thus, the combination of optimum parameters is evaluated. As can be inferred from Table 4, the optimum FoM is seen to be 3.77 × 10^−3^ which is higher than that of design number 3 which is 3.56 × 10^−3^. The level of confidence from the Taguchi prediction is observed to be 95.8%, which indicates that the Taguchi statistical method is a robust method to select for optimum combination of design parameters in micro-mixers.

## 5. Concluding Remarks

A computational study of micro-mixing in microchannel T-junction with wavy structure has been carried out together with the Taguchi statistical method to evaluate the significance of key design parameters with regards to the mixing performance, pumping power and figure of merit. The Taguchi method is found to be robust to determine the optimum combination of design parameters with the maximum relative error of less than 7%.

It has also been shown that, for high value chemical product such as in the pharmaceutical industry, optimization based on mixing index is suggested; the optimum design parameters are high wavy frequency, high wavy amplitude, narrow width and short channel cross-section, while for cheap value and mass production product, for which production cost is important, for example in food industries, optimization based on pumping power is recommended. The optimum design parameters are low frequency, low amplitude, wide and tall channel cross-section. On the other hand, one can also optimize the design based on the figure of merit for middle-value products by taking into account both mixing performance as well as pumping power, for which the parameters are the same as optimization based on pumping power. The results presented herein can aid engineers to determine the best design for micro-mixer performance. Future work will focus on more rigorous optimization procedures in order to alleviate the current limitation of discrete level optimization parameters. Possible coupling of optimization software with CFD software will also be explored.

## Figures and Tables

**Table 1:** Orthogonal array for L_9_ with four parameters and three level designs.

No.	Frequency (π)	Amplitude (mm)	Width (mm)	Height (mm)
1	2	0.5	0.25	0.25
2	2	1	0.5	0.5
3	2	2	1	1
4	5	0.5	0.5	1
5	5	1	1	0.25
6	5	2	0.25	0.5
7	10	0.5	1	0.5
8	10	1	0.25	1
9	10	2	0.5	0.25

**Table 2:** Physical and geometrical parameters.

Parameter	Value	Unit
Channel length	10	mm
Liquid density	998	kg/m^3^
Viscosity	1 × 10^−3^	kg/m∙s
Diffusivity	2.2 × 10^−9^	m^2^/s
Velocity inlet A	0.04	m/s
Velocity inlet B	0.04	m/s

**Table 3:** Numerical results of various combination of design factors.

No.	Mixing Index	Pressure Drop (Pa)	Figure of Merit
1	0.171	286.76	5.98 × 10^−4^
2	0.105	87.2	1.21 × 10^−3^
3	0.149	41.85	3.56 × 10^−3^
4	0.115	68.36	1.68 × 10^−3^
5	0.142	238.73	5.93 × 10^−4^
6	0.563	3747.13	1.50 × 10^−4^
7	0.203	103.58	1.96 × 10^−3^
8	0.582	3506.95	1.66 × 10^−4^
9	0.649	13,729	4.73 × 10^−5^

**Table 4:** Optimum combination of design factors.

Parameter	Mixing Index	Pressure Drop	Figure of Merit
Frequency	10	2	2
Amplitude	2	0.5	0.5
Width	0.25	1	1
Height	0.25	1	1
Optimized design	0.8	21.75	3.77 × 10^−3^
CI (%)	94.6%	93.8	95.8
